# Improving Organizational Commitment among Healthcare Employees in Angola: The Role of Psychological Capital and Perceived Transformational Leadership

**DOI:** 10.3390/healthcare12030326

**Published:** 2024-01-26

**Authors:** Rosa Lutete Geremias, Miguel Pereira Lopes, Ana Maria Sotomayor

**Affiliations:** 1 Lisbon Accounting and Business School, Instituto Politécnico de Lisboa, 1069-035 Lisbon, Portugal; amsotomayor@iscal.ipl.pt; 2 Centro de Administração e Políticas Públicas, 1300-663 Lisbon, Portugal; 3 Higher Institute of Social and Political Sciences, University of Lisbon, 1300-663 Lisbon, Portugal; mplopes@iscsp.ulisboa.pt; 4 Instituto Jurídico Portucalense, Universidade Portucalense, 4200-072 Porto, Portugal

**Keywords:** psychological capital, perceived transformational leadership, affective commitment, normative commitment, continuance commitment, Angola

## Abstract

While previous studies conducted in sub-Saharan African countries have focused on verifying standards of clinical care and assessing challenges faced by healthcare professionals, the present study fills a gap in the literature in that it explores the factors that may drive the organizational commitment of healthcare professionals in Angola. This study aimed to analyze the relationship between psychological capital and organizational commitment through perceived transformational leadership. Therefore, using the quantitative methodology, a self-report questionnaire was applied to 342 healthcare professionals (174 male, 168 female) from different public and private hospitals located in three large cities in Angola. The results confirmed that psychological capital is positively related to affective commitment and that perceived transformational leadership is a mediating variable of this relationship. Therefore, this study highlights the role of psychological capital and perceived transformational leadership in improving affective commitment in challenging environments.

## 1. Introduction

The last few decades have been marked by the need for healthcare systems to produce better results to drive social progress. For this reason, healthcare systems should contemplate certain changes in their work procedures to accommodate new challenges and ideas that allow for optimizing healthcare and responding to the needs of the population in different contexts [1]. Thus, the identification of healthcare professionals who perform their activities with a high level of commitment was considered a key point in previous research [2].

As advocated by [3], concerns related to the commitment of healthcare professionals have increased drastically, due to the work overload caused by the emergence of COVID-19. According to [4], with the appearance of COVID-19, the challenges that African countries face in terms of the quality of healthcare professionals worsened, due to the scarcity of resources channeled to the healthcare area. Overall, healthcare professionals in Africa have shown a low level of organizational commitment, which can be demonstrated by the frequent strikes of these professionals [5]. Angola is one of these African countries in which healthcare professionals have faced several challenges [6]. Angola is a sub-Saharan African country sharing borders with Namibia, Zambia, Congo, and the Democratic Republic of Congo.

The frightening increase in demand for healthcare requires committed healthcare professionals. Therefore, a high level of organizational commitment by healthcare professionals has implications for greater job satisfaction, reduced turnover, and absenteeism. On the other hand, a lower level of organizational commitment can contribute to a greater occurrence of medical errors, and thus negatively affect patient safety [7]. Although largely ignored in studies related to healthcare professionals in Africa, we argue that psychological capital may be of interest in improving the understanding of organizational commitment. Psychological capital has been identified in the literature as a predictor of different positive organizational outcomes [8]. On the other hand, [9] argue that employees’ commitment is an essential indicator to predict employee performance; therefore, the development of psychological capabilities is crucial.

Hospital managers have faced difficulties in maintaining committed professionals who can provide high-quality healthcare services in daily interaction with patients [10]. These difficulties encouraged different researchers to analyze the factors that can boost organizational commitment; however, studies carried out in Angola focused on verifying standards of clinical care and evaluating the challenges faced by healthcare professionals [6]. Therefore, understanding the factors that can drive the organizational commitment of healthcare professionals is important, as it allows us to investigate why there are high discrepancies in the quality standards of healthcare services.

For Donkor [11], the transformational leader can positively influence the achievement of organizational outcomes. Although there is no clear evidence of studies in the hospital context in Africa that have directly included employees’ perceptions of transformational leadership, researchers have been increasing interest in analyzing the link between psychological capital and organizational commitment [12]. However, we have seen some attempts to carry out studies that provide results related to the positive influence of psychological capital and perceived transformational leadership on the organizational commitment of teachers in Pakistan [13].

Our logic is based on studies that found evidence of the role of psychological capital and perceived transformational leadership in organizational commitment. Therefore, this study aimed to analyze the relationship between psychological capital and organizational commitment through the perceived transformational leadership of healthcare professionals in Angola. According to [14], a functional healthcare system requires that its professionals are motivated and committed to the organization. Therefore, we argue that the study of psychological capital and perceived transformational leadership can have theoretical and practical implications for the employee’s organizational commitment in Africa as a whole and Angola in particular. First, our study presented evidence that psychological capital is related to the affective commitment of healthcare professionals, even in contexts where there is a scarcity of financial resources allocated to improving the quality of healthcare professionals. Our work therefore contributes to the PsyCap literature by examining its implications in challenging contexts. Second, by analyzing perceived transformational leadership as the mechanism through which PsyCap affects affective commitment, our study contributes to explaining the role of transformational leadership in promoting positive outcomes [15]. This result responds to calls for more theoretical bases that allow understanding under which circumstances leadership can be an antecedent of psychological capital [16]. Finally, our results demonstrated that psychological capital is not related to continuance commitment. Therefore, our study contributes to relaunching the discussion about the relevance of the three-component model of organizational commitment [17].

This article is structured as follows. First, we define the variables under study, namely psychological capital, organizational commitment, and perceived transformational leadership, as well as the outline of the study’s hypotheses. Second, the methodological options and procedures are described, as well as the results. Third, we analyze the main results of the study, as well as a detailed description of the theoretical and practical implications. Finally, we present a brief conclusion of the study.

## 2. Theory and Hypotheses

### 2.1. Positive Psychological Capital

Psychological capital (PsyCap) is included in the Positive Organizational Behavior (POB) framework and has been identified as a higher-order construct that has a positive impact on individual development [18]. PsyCap incorporates certain positive psychological capabilities, such as self-efficacy, hope, resilience, and optimism, and has been widely considered a higher-order construct [19]. These psychological capabilities were included in psychological capital because they meet certain criteria: (1) they are based on theory and can be measured; (2) they have a positive impact on different results; and (3) it is open to development; therefore, it is state-like [20].

For [21], PsyCap is composed of four psychological capabilities, specifically: (1) self-efficacy: which is related to the individual confidence that allows the performance of challenging tasks with the necessary level of effort; (2) hope: associated with the perseverance necessary to achieve goals and allows redirecting paths to achieve success; (3) resilience: related to the individual capability to face obstacles or recover from adversity to achieve the desired success; (4) optimism: consists of making positive attributions or expectations when analyzing future events [22], presented evidence that attests to the convergent and discriminant validity of the Psychological Capital scale from other individual variables described in the literature, such as the “Big-five” personality characteristics.

The main psychological capabilities that make up psychological capital (self-efficacy, optimism, hope, and resilience) may increase the level of individual psychological capital, which can contribute to the effective execution and completion of tasks [23]. Therefore, psychological capital has been widely analyzed as a second-order construct, creating a synergistic motivational effect through the interaction of the four psychological capabilities [24]. For [25], psychological capital is a second-order construct that has been related to different positive results in the organizational field. The most recent meta-analysis in psychological capital research examined 244 studies during the period 2007–2020 and concluded that psychological capital positively affects different outcomes, such as work engagement, performance, and satisfaction [26].

In the context of healthcare, previous studies have demonstrated a positive relationship between psychological capital and the Management of Clinical Psychological Stressors [27], reward satisfaction [28], well-being [25], mental health [18], work engagement [29], and job satisfaction [30]. However, more studies are needed to understand the relationship more comprehensively between psychological capital and other positive outcomes, especially the organizational commitment of healthcare professionals.

### 2.2. Organizational Commitment

Organizational commitment is related to the employee’s desire to make efforts to remain in the organization, accepting its values, objectives, and principles [31]. According to [17], organizational commitment allows us to analyze the connection that the employee maintains with their organization, as well as the nature of the employment relationship. Therefore, the conceptualization of organizational commitment found in the literature is based on certain assumptions, such as the existence of a psychological bond that the employee needs to maintain with their organization and identification with the organization’s objectives and values [32].

According to [33], organizational commitment is based on a three-component model that includes affective commitment, normative commitment, and continuance commitment. Affective commitment has been defined as an emotional bond that allows for greater employee involvement with the organization [34]. Normative commitment is related to the employee’s permanence in a given organization motivated by a feeling of obligation/moral duty [35]. On the other hand, continuance commitment consists of the employee remaining in the organization motivated by the high costs associated with leaving [36].

In the area of healthcare, the commitment of its employees has been considered a key pillar of society, considering that it boosts well-being and satisfaction at work, essential factors for the provision of healthcare with quality standards essential for patient safety [37]. Therefore, healthcare organizations need to promote a culture that allows their employees to be strongly committed to previously defined values, objectives, and policies [2]. According to [12], analyzing healthcare professionals’ perceptions of organizational commitment is crucial for better performance at work.

The healthcare professionals’ commitment has been described as a crucial factor in the success of healthcare organizations, considering that it helps the organization achieve its defined objectives through the quality of healthcare services, and promoting the efficiency and effectiveness of its processes [37]. According to [11], organizational commitment can be considered a valuable variable for both the employee and the organization, given that an employee with a high level of organizational commitment identifies with the organization’s values, and tends to perform required tasks successfully.

### 2.3. Perceived Transformational Leadership

Transformational leadership has been conceptualized as a behavioral process that contributes to improving the performance of your subordinates through the process of sharing the leader’s vision of the future [38]. The transformational leader tends to emphasize achieving the organization’s objectives and developing their followers, leaving their personal interests behind [15]. Therefore, the transformational leader can directly influence the performance and commitment of their subordinates, which can help to better predict the success of the organization [39].

For [38], the transformational leader shares the organization’s visions and objectives with their subordinates, allowing these employees to have information about the organization’s development, as well as the paths the organization should take, thus adding value to the organization and its stakeholders. Therefore, transformational leaders consider their followers’ ideas in the policy-making process while helping them improve their performance [40]. According to [15], transformational leadership is crucial for the success of organizations, considering that it contributes to reducing followers’ turnover, thus leading to greater organizational commitment.

The study carried out by [41] highlighted the positive relationships between transformational leadership and the achievement of positive results by its subordinates, but it also recognized the barriers related to exercising transformational leadership in challenging contexts. For [42], transformational leaders are those who, in adverse circumstances, seek alternative ways of working, and make effective changes to obtain positive results, thus becoming the key determinant of organizational results.

In the healthcare field, transformational leadership has been considered a crucial element, given that it helps healthcare professionals provide patients with well-coordinated and integrated services [43]. For [38], the decisions of transformational leaders have important implications for performance, job satisfaction, and talent retention. Therefore, transformational leadership is relevant considering that it contributes to greater employee commitment while allowing patients to be provided with an environment conducive to improving healthcare [44].

### 2.4. Psychological Capital and Organizational Commitment

Psychological capital has been identified in the literature as a driving force that has a positive impact on different organizational outcomes, such as organizational commitment [20]. Different studies have shown a positive relationship between psychological capital and organizational commitment. For example, [35] carried out a study with 106 South African employees and the results showed that psychological capital positively affects organizational commitment. According to [45], psychological capital, affective commitment, and normative commitment share the same positive nature; therefore, there is a greater probability of these variables showing a positive relationship.

An empirical study conducted in the healthcare industry by [46] demonstrated that the psychological capital of nurses with a high level of stress caused by long working hours was positively related to their organizational commitment. For [45], the development of psychological capital can contribute to greater organizational commitment, and thus contribute to organizational success. In this context, employees with a high level of psychological capital tend to be more committed to the organization [47].

Ref. [48] argue that studying the psychological capital of healthcare professionals is crucial, considering that it helps these professionals deal with stress factors caused by little work autonomy, as well as certain behavioral and healthcare problems of patients. Furthermore, the analysis of the psychological capital of healthcare professionals in Angola can help employees improve their level of organizational commitment, and thus be able to face adverse circumstances due to the scarcity of financial, material, and human resources, resulting from the reduced healthcare budget [6]. This evidence leads us to formulate the following hypothesis:

**H1:** *psychological capital is positively related to organizational commitment*;

**H1a:** *psychological capital is positively related to affective commitment*;

**H1b:** *psychological capital is positively related to normative commitment*;

**H1c:** *psychological capital is positively related to continuance commitment*;

### 2.5. Psychological Capital and Perceived Transformational Leadership

The relationship between psychological capital and perceived transformational leadership was considered relevant in previous studies [49]. According to [50], employees with greater self-efficacy and optimism when performing their tasks tend to identify transformational leadership behaviors that manifest themselves through mentoring, coaching, and delegation of challenging tasks. Furthermore, employees who are highly effective in carrying out tasks to achieve defined objectives tend to perceive their leaders as transformational during the process of sharing the organization’s results [51]. The study carried out by [52] included 121 sales representatives from a pharmaceutical company in Australia and concluded that employees who developed hopeful behaviors demonstrated greater awareness of the support they received from transformational leaders. 

In the healthcare sector, the study carried out by [53] found empirical evidence of the relationship between resilience and perceived transformational leadership. For [54], transformational leadership is the leadership style that presents characteristics that can be positively related to the different psychological capabilities of healthcare professionals. However, there is little empirical evidence that analyzes the influence of psychological capital on the perceived transformational leadership of healthcare professionals in Africa. 

Previous empirical research using cross-sectional studies supported the opposite relationship proposed in this study, that is, the perceived transformational leadership is what influences the development of employees’ psychological capabilities [49,50]). These previous conclusions can be explained by the fact that it is not possible in cross-sectional studies to attribute directionality to the relationships defined between the different variables [55]. Given this, we hypothesize that:

**H2:** *Psychological capital is positively related to perceived transformational leadership*.

### 2.6. Perceived Transformational Leadership and Organizational Commitment

Transformational leadership has been conceptualized as one of the factors that contribute to the development of different positive outcomes [38]. According to [40], transformational leaders tend to consider the needs of their followers and encourage the development of new approaches to performing tasks and solving problems. These factors motivate employees to be more involved with their work, which has implications for increasing their organizational commitment.

Previous studies have highlighted empirical evidence that attests to the positive relationship between perceived transformational leadership and organizational commitment in different contexts, especially in the healthcare field [40]. According to [56], transformational leaders tend to prioritize satisfying the needs of their followers, which contributes to strong organizational commitment, and consequently a long stay of employees in the organization.

The study carried out by [57] presented empirical evidence of the positive influence of perceived transformational leadership on the organizational commitment of 580 employees in the tourism sector. However, there have been few attempts to carry out studies that would allow us to analyze the influence of perceived transformational leadership on the organizational commitment of healthcare professionals in southern Africa. Despite this, there is evidence of the link between organizational commitment and perceived transformational leadership in Africa, specifically among healthcare professionals in Ethiopia. Therefore, this empirical evidence allows us to formulate the following hypotheses:

**H3:** *perceived transformational leadership is positively related to organizational commitment*;

**H3a:** *perceived transformational leadership is positively related to affective commitment*;

**H3b:** *perceived transformational leadership is positively related to normative commitment*;

**H3c:** *perceived transformational leadership is positively related to continuance commitment*.

### 2.7. The Mediating Role of Perceived Transformational Leadership

Previous research has highlighted the relevance of the mediating role of perceived transformational leadership for understanding different positive results in the organizational field. In [58,59], it was argued that employees who developed psychological capital perceived transformational leadership differently from employees with low psychological capital. Previous studies have also concluded that the psychological capital of healthcare professionals is positively related to perceived transformational leadership and may boost employee retention [40].

According to [60], psychological capital has been applied in the healthcare field to help professionals increase organizational commitment and overcome exhaustion. Thus, the development of psychological capital allows for a better perception of the leadership style used, which can contribute to achieving different positive results in the organizational field [47]. For these reasons, we argue that employees who develop psychological capital and perceive their leaders as transformational can have greater organizational commitment. As such, we hypothesize that:

**H4:** *Perceived transformational leadership mediates the relationship between psychological capital and organizational commitment;*

**H4a:** *Perceived transformational leadership mediates the relationship between psychological capital and affective commitment;*

**H4b:** *Perceived transformational leadership mediates the relationship between psychological capital and normative commitment;*

**H4c:** *Perceived transformational leadership mediates the relationship between psychological capital and continuance commitment.*

**Figure 1:**
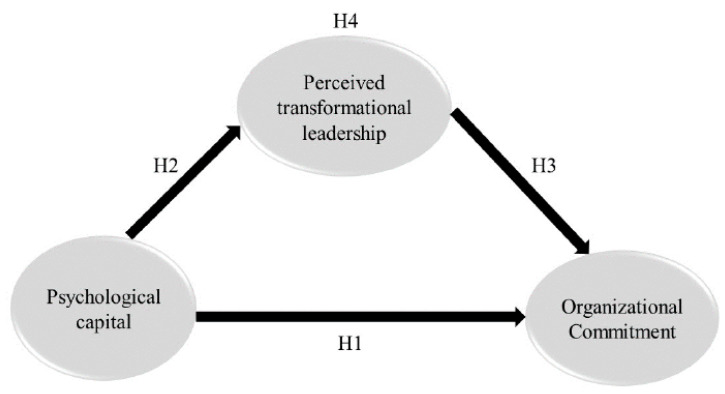
A conceptual model of the relationship between psychological capital, perceived transformational leadership and organizational commitment.

Figure 1 presents the conceptual model of the relationship between psychological capital and organizational commitment through perceived transformational leadership.

## 3. Materials and Methods

### 3.1. Design

In the present study, we used the quantitative methodology, with a correlational and descriptive design to explore the relationships between the main variables under study. To this end, a cross-sectional field survey of healthcare professionals in Angola was carried out. The sample was selected using a non-probabilistic convenience technique, in which the selected participants were expected to provide healthcare in public and private hospitals in the three provinces of Angola.

### 3.2. Participants

The participants were healthcare professionals from different public and private hospitals located in three large cities in Angola, specifically Luanda, Benguela, and Huíla. These provinces were selected from a list of 18 provinces in Angola. It is important to note that these three provinces were chosen due to their greater population density, as they are where hospitals receive the highest inflow of patients daily. Therefore, employees in these hospitals are typically subjected to a high workload, which has caused high physical and emotional exhaustion [6]. 

During October 2023, links were made available on the online platform, the expectation was to reach 500 employees. However, 342 questionnaires were received, so the estimated acceptance rate is 68%. In total, 342 questionnaires from 30 public and private healthcare institutions (ranging from 10 to 15 healthcare professionals per institution) have been validated. The number of study participants (342) exceeds the number considered appropriate (200) for carrying out structural equation modeling considering the maximum likelihood estimation, as suggested by [61].

Considering the data obtained, we highlight that 51% of the participants were men, with an average age of 34 years (*SD* = 5.35). The most significant specialty areas were intensive care (23%), nursing (16%), and ophthalmology (11%). Concerning educational qualifications, it was found that 70% of participants have bachelor’s degrees, 19% have a master’s degree and 11% are attending a doctoral course. Furthermore, it is important to highlight that 33% had been working for 5 years in the organization, 25% had been working for 6 years, and 21% had been working for 4 years.

### 3.3. Procedures

All participants voluntarily completed the questionnaire available on the online platform and marked the field related to informed consent before beginning to complete the survey. It is important to note that we followed standard ethical procedures, such as the communication that must be made to participants about confidentiality in the collection and processing of data, as well as the voluntary participation of all participants. Additionally, clarifications were provided that the questionnaire was anonymous; therefore, it would not be possible to identify the participants individually. On the other hand, all requests for clarification of doubts raised by participants when completing the survey were addressed.

### 3.4. Measures

Psychological capital. We used the 24-item version of the questionnaire developed by [21]. The scale consists of four subscales with six items each, corresponding to positive psychological capabilities evaluating, respectively, optimism (e.g., “At work, I always look at the positive side of things”); hope (e.g., “I can think of many ways to achieve my current goals at work”); self-efficacy (e.g., “I feel confident when contributing to discussions about the Institution’s strategy”); resilience (e.g., “In general, I easily overcome the most stressful things at work”). All the responses were given on a six-point Likert scale, from (1) “Strongly Disagree” to (6) “Strongly Agree”. The 24-item positive psychological capital scale presented in the original study has a Cronbach’s α of 0.89.

Perceived transformational leadership. We used the version of the questionnaire by [62], which was validated for Portuguese by [63]. This questionnaire consists of 20 items (for example: “My team leader is not afraid to break routines to find different ways of doing things”), and the value of Cronbach’s Alpha coefficient is 0.92. The response scale used is a five-point Likert type, from (1) “Totally Disagree” to (5) “Totally Agree”.

Organizational commitment. We used the scale developed and validated by [33], consisting of 3 subscales and 19 items, specifically: affective commitment with 6 items (for example: “I feel this company’s problems as if they were mine”); the Cronbach’s Alpha coefficient presented by the authors is 0.90. Normative Commitment consists of 6 items (for example: “I feel that I have a great duty to this company”), and the value of Cronbach’s Alpha coefficient mentioned by the authors is 0.89. Continuance Commitment has 7 items (for example: “A lot of my life would be affected if I decided to leave this company at this moment”), the Cronbach’s Alpha coefficient reported by the authors is 0.90. The answers were given on a 7-point Likert scale, in which (1) corresponds to “Totally Disagree” and (7) “Totally Agree.”

The scales used in this study were previously translated into Portuguese according to rules widely used and validated in empirical studies, such as the translation/retroversion method, e.g., [64,65]. The translated versions were carefully compared with the original scale by an English-speaking native and a Portuguese-English linguistic lecturer.

### 3.5. Data Analysis

In the present study, the analysis took place in different phases. Firstly, the SPSS software (v.27) was used to analyze the descriptive statistics of the variables under study. Second, confirmatory factor analysis was performed using Amos (v.27) to examine the factor structure of psychological capital, perceived transformational leadership, and organizational commitment.

To evaluate the goodness fit of the scales under study, we used the following acceptability parameters recommended by [61]: Chi-Square (χ^2^): *p*-value ≤ 0,05; Tucker–Lewis Index (TLI): ≥0.90; Comparative Fit Index (CFI): ≥0.90; Goodness of Fit Index (GFI): ≥0.90; Standardized Root Mean Square Residual (SRMR): ≤0.08; Root Mean Square Error of Approximation (RMSEA): ≤0.08. On the other hand, to assess the reliability of the scales used in the present study, we used the SPSS software to calculate Cronbach’s alpha coefficients.

### 3.6. Measurement Validity

We validated the factorial structure of the constructs under study by carrying out confirmatory factor analysis. The reliability of the individual items and the factor weights found allowed us to assess the quality of the model fit. Additionally, we checked the convergent validity, that is, its composite reliability, following the suggestions of [66]. We performed confirmatory factor analysis using the Amos software for all constructs under analysis. Psychological capital as a second-order construct presents adequate adjustment indexes (χ^2^ (231) = 315.386, ρ < 0.001; TLI = 0.902; CFI = 0.918; GFI = 0.933; SRMR = 0.051; RMSEA = 0.033). Cronbach’s Alpha for the Positive Psychological Capital dimension was 0.72.

Additionally, the perceived transformational leadership scale presents adequate adjustment indexes (*χ^2^* (13) = 29.892, ρ < 0.001; TLI = 0.982; CFI = 0.992; GFI = 0.978; SRMR = 0.051; RMSEA = 0.062). Cronbach’s alpha of perceived transformational leadership was 0.93. The Organizational Commitment Scale presents adequate adjustment indexes (*χ^2^* (116) = 180.075, ρ < 0.001; TLI = 0.940; CFI = 0.959; GFI = 0.883; SRMR = 0.079; RMSEA = 0.064). Cronbach’s alpha of Organizational Commitment was 0.86.

## 4. Results

### 4.1. Descriptive Statistics

Table 1 describes the results related to means, standard deviations, as well as values that reflect the internal consistencies of the variables under study (Cronbach’s alphas in parentheses). Additionally, the results of Pearson correlations between the constructs under study were presented. As expected, psychological capital positively correlates with perceived transformational leadership and organizational commitment. The results in Table 1 demonstrate that all Cronbach’s alpha values are adequate. The model that presents the indirect effect of perceived transformational leadership on the relationship between psychological capital and organizational commitment was considered adjusted for the 342 participants under study. The proposed model presents appropriate adjustments (χ^2^ (42) = 98.446, ρ < 0.001; TLI = 0.922; CFI = 0.956; GFI = 0.909; SRMR = 0.079; RMSEA = 0.037).

On the other hand, to analyze the explanatory power of the number of participants in this study, we resorted to the power analysis program for structural equation modeling. For this purpose, we used the following data: anticipated effect of 0.144; probability level of 0.05; and statistical power level of 0.80, as recommended by [67]. The results obtained demonstrated that 294 participants would be adequate for the reliability of the results presented. Therefore, we argue that the number of participants selected for the present study provides adequate explanatory power for the results.

### 4.2. Assessing Common Method Bias

According to [68], the potential problem of common method bias arises from the data collection process using a single source, also considering a specific moment for the data collection. Therefore, different authors such as [69] have recommended the use of statistical “remedies” to minimize this problem. To this end, we used the common method factor to assess whether common method bias could have negatively influenced the results presented. The results obtained demonstrated that the value obtained by the common method factor was 16.32 percent. According to [70], the value of the common method factor should not exceed 25 percent. Therefore, we argue that common method bias is not a concern for the reliability of the results presented.

### 4.3. Hypothesis Tests

To analyze the mediating role of perceived transformational leadership in the relationship between psychological capital and organizational commitment, we used the approach proposed by [71]. First, we analyze the link between psychological capital and the three dimensions of organizational commitment. Second, we make the connection between psychological capital and perceived transformational leadership. Third, we analyze the relationship between perceived transformational leadership and the three dimensions of organizational commitment. On the other hand, we used the [72], to check the perceived transformational leadership’s potential mediating role.

The results show that psychological capital has a positive and statistically significant influence on affective commitment (β = 0.29; ρ < 0.001), which supports H1a. The positive influence of psychological capital on normative commitment was supported (β = 0.28; ρ < 0.001), confirming H1b. However, contrary to our predictions, the result does not support the positive influence of psychological capital on continuance commitment (β = 0.03; ρ = 0.815), rejecting H1c. For this reason, we need to partially support H1.

Psychological capital was shown to influence perceived transformational leadership significantly and positively (β = 0.59; ρ < 0.001), supporting H2. Perceived transformational leadership is significantly and positively associated with affective commitment (β = 0.12; ρ < 0. 001), which supports H3a. The positive influence of perceived transformational leadership on normative commitment was not confirmed (β = 0.06; ρ = 0.230), causing us to reject H3b. Additionally, the result does not support the positive influence of perceived transformational leadership on continuance commitment (β = 0.09; ρ = 0.106), rejecting H3c. This result partially supports H3. 

**Figure 2:**
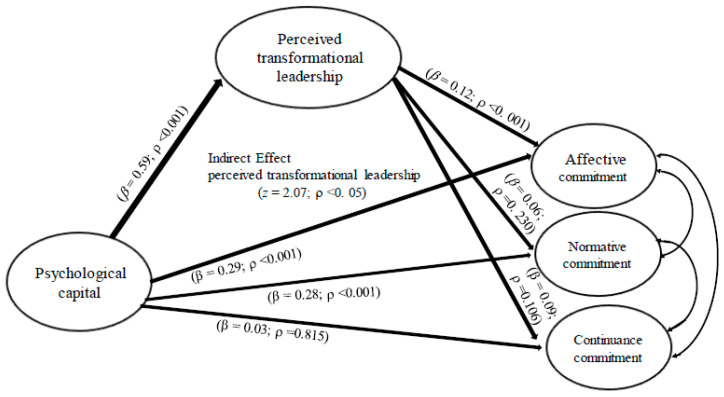
Final model.

It is important to note that all the conditions presented for carrying out the mediation test proposed by [71] were met only for affective commitment. This result leads us to reject H4b,c. Therefore, we perform the Sobel test [72] as a means of further examining evidence for the mediating role of perceived transformational leadership in the relationship between psychological capital and affective commitment. The results of the Sobel test supported that perceived transformational leadership fully mediates the relationship between psychological capital and affective commitment (z = 2.07; ρ < 0.05), which supports H4a. This result allows us to partially confirm H4. Therefore, Figure 2 presents all the results of the hypothesis test.

## 5. Discussion

The main purpose of this study was to analyze the relationship between psychological capital and organizational commitment through perceived transformational leadership. The results obtained corroborated the positive link between psychological capital and affective commitment (H1a). These results lead us to argue that the development of psychological capital contributes to healthcare employees being emotionally connected to their organization. This result is in line with the previous literature indicating that without the development of psychological capital, healthcare professionals will have difficulty implementing best practices to achieve different positive results, and thus develop an emotional connection with the organization when challenging changes require new approaches [37].

On the other hand, different studies indicate that healthcare professionals are exposed to challenging and uncertain environments; therefore, the development of psychological capital becomes crucial for the affective commitment of these professionals [2,30]). Furthermore, previous research has shown positive and significant results between psychological capital and affective commitment [73]. This seems to happen because employees who develop psychological capital regardless of the environment in which they are inserted face problems or resort to the process of positive adaptation to overcome difficulties, which may result in an emotional connection with the organization.

The relationship between psychological capital and normative commitment was also confirmed (H1b). This relationship seems to happen because, healthcare professionals with high levels of psychological capital rarely give up when faced with challenges and adverse situations and seek alternatives to solve different problems due to their positive vision and sense of opportunity, even if their stay in the organization is motivated by a feeling of obligation [24,74]. Moreover, these results are consistent with previous studies that highlight the influence of psychological capital on normative commitment [75].

The results presented did not confirm the positive influence of psychological capital on continuance commitment (H1c). This seems to happen because contrary to what was historically believed, continuance commitment can be strongly influenced by the distribution of extrinsic rewards, so employees tend to develop continuance commitment in organizations where rewards are offered in exchange for their efforts [76]. Continuance commitment might be conditioned by active stimulation at an early stage, something that may not be related to psychological capital. Therefore, psychological capital may, eventually, be important in a later stage when healthcare organizations will be able to allocate rewards compatible with the healthcare professionals’ performance. However, this possibility needs to be tested.

Our second H(H2) was supported, relating psychological capital and perceived transformational leadership. These results are consistent with previous studies, e.g., [77], which have focused on psychological capital and its relationship to leadership [49], arguing that employees with a high level of psychological capital tend to give the leader opportunities to reflect on their actions; therefore, these leaders develop the ability to recalibrate their inner resources, as well as the behaviors required for inspiring and effective leadership. Thus, we argue that the development of psychological capital can act as a lever to approach leaders with their professional ideals, such as inspiration for a vision.

The relationship between perceived transformational leadership and affective commitment was also confirmed (H3a). This result confirms what has been demonstrated in previous studies, such as the study carried out by [57]. As reported by [13], this relationship occurs because a transformational leader tends to encourage his employees to exceed expected performance, which contributes to the development of affective commitment. In a context in which healthcare professionals face many problems caused by the scarcity of resources channeled to the healthcare area, the transformational leader, by intellectually stimulating his followers, and by sharing his vision of the future, may positively influence the employee’s desire to remain in the organization.

Contrary to what was defined, the results presented did not confirm the positive influence of perceived transformational leadership on normative commitment and continuance commitment (H3b,c). As pointed out by [78], given normative commitment and continuance commitment are linked to involvement based on moral duty, on the one hand, and the high costs associated with leaving, on the other hand, employees may be indifferent to the leader’s actions. Despite this, the lack of a positive influence of perceived transformational leadership on continuance commitment is consistent with studies developed by [79].

However, the absence of the positive influence of perceived transformational leadership on normative commitment is an unexpected finding. Could it be possible that affective commitment is the only dimension of organizational commitment that may be influenced by perceived transformational leadership? This seems to happen because, contrary to what has been widely pointed out, e.g., [40], healthcare professionals who have higher levels of affective commitment are more likely to carry out their work with passion, despite adverse circumstances [80].

The mediating role of perceived transformational leadership in the relationship between psychological capital and affective commitment was supported (H4a). This result seems to corroborate the study carried out by [81], in which the authors argue that employees with a high level of psychological capital are more likely to perceive their leader as visionary and motivating, and consequently tend to be committed to the organization. According to [38], transformational leadership plays a crucial role in developing affective commitment in the organizational field.

Finally, it is important to discuss the challenges related to developing psychological capital in countries with emerging economies, as highlighted by [82]. For [26], the difficulty related to developing psychological capital can be overcome, as psychological capital is a positive individual characteristic that is malleable when there is a commitment to empowering individuals. Therefore, it is important to focus on training with certain interventions to develop psychological capital. The study carried out by [83], used a double sample of students and managers, measuring the psychological capital of participants one week before and one week after a workshop, and the results obtained demonstrated significant improvements in levels of psychological capital and performance of these professionals.

### 5.1. Limitations and Future Directions

The present study has some limitations. The present study analyzed transformational leadership through followers’ perceptions. This option may have affected the results obtained, considering that followers may not have an accurate perception of aspects related to leadership [63,84]. Therefore, future studies may consider the possibility of analyzing leadership from the leader’s point of view.

Another limitation is related to the potential common method variance problem since we used the same source to collect data for all variables under study at the same point in time. However, we used the common method factor as one of the statistical “remedies” to determine whether common method bias could have affected the reliability of the results presented. Future studies could resort to other alternatives to minimize the problem of common method bias, such as using at least two-time points of data collection, as suggested by [64].

Finally, it would be important to consider the use of heterogeneous samples in future studies. As pointed out by [85], healthcare professionals are more likely to develop affective commitment, which might be a constraint for the generalizability of this study.

### 5.2. Theoretical and Practical Implications

Our study contributes to the literature in several ways. Firstly, we provide evidence that the psychological capital of healthcare professionals in Angola can influence affective commitment and normative commitment, which means that the development of psychological capital contributes to achieving positive outcomes, even in challenging environments. Therefore, this study suggests that it is important to promote the development of the psychological capital of healthcare professionals. Furthermore, this study may raise awareness among managers and public policymakers about the need to carry out training sessions to develop psychological capital.

Our second contribution comes from the notion widely defended in the literature that perceived transformational leadership is what influences psychological capital and not the other way around. These findings fill an important gap in the field of psychological capital and are in line with the concern presented by [16], related to the absence of a strong theoretical basis to support why leadership should be an antecedent of psychological capital.

Finally, another contribution is related to the fact that perceived transformational leadership did not influence normative commitment and continuance commitment. These findings contradict empirical evidence that shows the influence of perceived transformational leadership on all dimensions of organizational commitment, e.g., [40]. Given this, managers or other individuals potentially perceived as leaders may need to make an additional effort to promote the affective commitment of their employees.

## 6. Conclusions

The main objective of this study was to analyze the relationship between psychological capital and organizational commitment through perceived transformational leadership. Knowing more about the factors that drive the organizational commitment of healthcare professionals in Angola is relevant, as it is considered an indicator of organizational performance since committed professionals contribute to effective patient treatment [5,7].

The results presented demonstrated that psychological capital is positively related to two components of organizational commitment, namely affective commitment, and normative commitment. Furthermore, we found evidence that perceived transformational leadership has an indirect effect on the relationship between psychological capital and affective commitment. Therefore, the present study emphasizes the efforts that should be considered to promote organizational commitment among healthcare professionals in Angola. In particular, the importance of psychological capital and perceived transformational leadership should not be underestimated by professionals, managers, and political decision-makers interested in improving levels of organizational commitment.

## Figures and Tables

**Table 1:** Means, standard deviations, and correlations between study variables.

Study Variables	*M*	*SD*	1	2	3	4	5	6
1. Psychological Capital	4.84	0.61	−0.89					
2. Perceived transformational leadership	4.01	1.21	0.299 **	−0.92				
3. Organizational Commitment	4.3	0.93	0.132 *	0.115 *	−0.9			
4. Affective commitment	3.99	1.21	0.145 **	0.119 *	0.884 **	−0.89		
5. Normative commitment	4.57	1.08	0.162 **	0.065	0.713 **	0.509 **	−0.78	
6. continuance commitment	4.34	1.21	0.013	0.087	0.778 **	0.576 **	0.237 **	−0.88

N = 342. Cronbach’s αs (in parentheses). *. The correlation is significant at the 0.05 level (2-tailed). **. The correlation is significant at the 0.01 level (2-tailed).

## Data Availability

All data will be made available by the authors, without undue reservation.

## References

[1] Apore G.N., Asamoah E.S. (2019). Emotional intelligence, gender and transformational leadership among nurses in emerging economies. Leadersh. Health Serv..

[2] Arage S., Daba D., Dessalegn A. (2022). Organizational commitment of health professionals and associated factors in primary healthcare facilities of Addis Ababa, Ethiopia: A multi-center cross-sectional study. Front. Public Health.

[3] Dai B., Akey-Torku B. (2020). The Influence of Managerial Psychology on Job Satisfaction among Healthcare Employees in Ghana. Healthcare.

[4] Amu H., Dowou R., Saah F., Efunwole J., Bain L., Tarkang E. (2022). COVID-19 and Health Systems Functioning in Sub-Saharan Africa Using the “WHO Building Blocks”: The Challenges and Responses. Front. Public Health.

[5] Muthuri R.N., Senkubuge F., Hongoro C. (2020). An Investigation of Healthcare Professionals’ Motivation in Public and Mission Hospitals in Meru County, Kenya. Healthcare.

[6] Gyeltshen D., Musa S.S., Amesho J.N., Ewelike S.C., Bayoh A.V., Al-Sammour C., Camua A.A., Lin X., Lowe M., Ahmadi A. (2021). COVID-19: A novel burden on the fragile health system of Angola. J. Glob. Health.

[7] Fantahun B., Dellie E., Worku N., Debie A. (2023). Organizational commitment and associated factors among health professionals working in public hospitals of southwestern Oromia, Ethiopia. BMC Health Serv. Res..

[8] Geremias R.L., Lopes M.P., Soares A.E. (2020). Enhancing Internal Learning in Teams: The Role of Network Centrality and Psychological Capital of Undergraduate Students. Front. Psychol..

[9] Lee M., Kim B. (2023). Effect of Employee Experience on Organizational Commitment: Case of South Korea. Behav. Sci..

[10] De las Heras-Rosas C., Herrera J., Rodríguez-Fernández M. (2021). Organisational Commitment in Healthcare Systems: A Bibliometric Analysis. Int. J. Environ. Res. Public Health.

[11] Donkor F., Dongmei Z., Sekyere I. (2021). The Mediating Effects of Organizational Commitment on Leadership Styles and Employee Performance in SOEs in Ghana: A Structural Equation Modeling Analysis. SAGE Open.

[12] Ruvimbo T.S., Hlanganipai N. (2016). Organisational commitment and job retention among nurses in a South African setting: An exploratory study. J. Psychol. Afr..

[13] Saadat U.R., Ali A., Habib M., Ali N., Shah M. (2023). Mediating Role of Psychological Capital In The Relationship between Transformational Leadership And Organizational Commitment. J. Posit. Sch. Psychol..

[14] Bafei S., Chen J., Qian Y., Yuan L., Zhou Y., Sambou M., Walker A.N., Li W., Liu S. (2023). The Association between Burnout, Social Support, and Psychological Capital among Primary Care Providers in Togo: A Cross-Sectional Study. Medicina.

[15] Eberly M.B., Bluhm D.J., Guarana C., Avolio B.J., Hannah S.T. (2017). Staying after the storm: How transformational leadership relates to follower turnover intentions in extreme contexts. J. Vocat. Behav..

[16] Wu W.-Y., Nguyen K.-V.H. (2019). The antecedents and consequences of psychological capital: A meta-analytic approach. Leadersh. Organ. Dev. J..

[17] Khan A., Rainayee R.A. (2022). Organisational Commitment: Conceptualization, Measurement, and Scale validation. Int. J. Sci. Technol. Manag..

[18] Luthans F., Broad J.D. (2020). Positive Psychological Capital to Help Combat the Mental Health Fallout from the Pandemic and VUCA Environment. Organ. Dyn..

[19] Alessandri G., Consiglio C., Luthans F., Borgogni L. (2018). Testing a dynamic model of the impact of psychological capital on work engagement and job performance. Career Dev. Int..

[20] Luthans F., Youssef-Morgan C.M. (2017). Psychological Capital: An Evidence-Based Positive Approach. Annu. Rev. Organ. Psychol. Organ. Behav..

[21] Luthans F., Avolio B.J., Avey J.B., Norman S.M. (2007). Positive Psychological Capital: Measurement and Relationship with performance and satisfaction. Pers. Psychol..

[22] Newman A., Ucbasaran D., Zhu F., Hirst G. (2014). Psychological capital: A review and synthesis. J. Organ. Behav..

[23] Parent-Rocheleau X., Bentein K., Simard G. (2020). Positive together? The effects of leader-follower (dis)similarity in psychological capital. J. Bus. Res..

[24] Broad J.D., Luthans F. (2020). Positive resources for psychiatry in the fourth industrial revolution: Building patient and family focused psychological capital (PsyCap). Int. Rev. Psychiatry.

[25] Youssef-Morgan C.M., Luthans F. (2015). Psychological capital and well-being. Stress Health J. Int. Soc. Investig. Stress.

[26] Loghman S., Quinn M., Dawkins S., Woods M., Om Sharma S., Scott J. (2023). A Comprehensive Meta-Analyses of the Nomological Network of Psychological Capital (PsyCap). J. Leadersh. Organ. Stud..

[27] Caponnetto P., Platania S., Maglia M., Morando M., Gruttadauria S., Auditore R., Ledda C., Rapisarda V., Santisi G. (2022). Health Occupation and Job Satisfaction: The Impact of Psychological Capital in the Management of Clinical Psychological Stressors of Healthcare Workers in the COVID-19 Era. Int. J. Environ. Res. Public Health.

[28] Shelton S.A., Renard M. (2015). Correlating nurses’ levels of Psychological Capital with their reward preferences and reward satisfaction. SA J. Ind. Psychol..

[29] Zhang M., Chen H., Wang N., Li Y., Li X., Liu Y. (2023). The mediating role of job satisfaction between psychological capital and work engagement among Chinese nurses during COVID-19 outbreak: A comparative study between nurse specialists and general nurses. Front. Psychiatry.

[30] Alan H., Polat S., Tiryaki S.H. (2022). The role of psychological capital in the relationship between nurses’ job satisfaction and turnover intention. Perspect. Psychiatr. Care.

[31] Herrera J., De Las Heras-Rosas C. (2021). The Organizational Commitment in the Company and Its Relationship with the Psychological Contract. Front. Psychol..

[32] Ng T.W. (2015). The incremental validity of organizational commitment, organizational trust, and organizational identification. J. Vocat. Behav..

[33] Meyer J., Allen N. (1991). A three-component conceptualization of organizational commitment. Hum. Resour. Manag. Rev..

[34] DiPietro R.B., Moreo A., Cain L. (2019). Well-being, affective commitment and job satisfaction: Influences on turnover intentions in casual dining employees. J. Hosp. Mark. Manag..

[35] Simons J.C., Buitendach J.H. (2013). Psychological capital, work engagement and organisational commitment amongst call centre employees in South Africa. SA J. Ind. Psychol..

[36] Choi D., Oh I.-S., Colbert A.E. (2015). Understanding organizational commitment: A meta-analytic examination of the roles of the five-factor model of personality and culture. J. Appl. Psychol..

[37] Al-Haroon H., Al-Qahtani M.F. (2020). Assessment of Organizational Commitment among Nurses in a Major Public Hospital in Saudi Arabia. J. Multidiscip. Heal..

[38] Chen C., Ding X., Li J. (2022). Transformational Leadership and Employee Job Satisfaction: The Mediating Role of Employee Relations Climate and the Moderating Role of Subordinate Gender. Int. J. Environ. Res. Public Health.

[39] Kloutsiniotis P., Mihail D., Mylonas N., Pateli A. (2022). Transformational Leadership, HRM practices and burnout during the COVID-19 pandemic: The role of personal stress, anxiety, and workplace loneliness. Int. J. Hosp. Manag..

[40] Iqbal K., Fatima T., Naveed M. (2019). The Impact of Transformational Leadership on Nurses’ Organizational Commitment: A Multiple Mediation Model. Eur. J. Investig. Health Psychol. Educ..

[41] Ramsey J.R., Rutti R.M., Lorenz M.P., Barakat L.L., Sant’anna A.S. (2017). Developing global transformational leaders. J. World Bus..

[42] Mdletshe N., Nzimakwe T.I. (2023). An analysis of the influence of transformational leadership in a taxation organisation in the KwaZulu-Natal region. J. Contemp. Manag..

[43] Kiwanuka F., Nanyonga R.C., Sak-Dankosky N., Muwanguzi P.A., Kvist T. (2020). Nursing leadership styles and their impact on intensive care unit quality measures–An Integrative review. J. Nurs. Manag..

[44] Sfantou D., Laliotis A., Patelarou A., Sifaki-Pistolla D., Matalliotakis M., Patelarou E. (2017). Importance of Leadership Style towards Quality of Care Measures in Healthcare Settings: A Systematic Review. Healthcare.

[45] Sahoo B.C., Sia S.K. (2015). Psychological Capital and Organisational Commitment: Nature, Structure and Relationship in an Indian Sample. Asia-Pac. J. Manag. Res. Innov..

[46] Zhou J., Yang Y., Qiu X., Yang X., Pan H., Ban B., Wang W. (2018). Serial multiple mediation of organizational commitment and job burnout in the relationship between psychological capital and anxiety in Chinese female nurses: A cross-sectional questionnaire survey. Int. J. Nurs. Stud..

[47] Dawkins S., Martin A., Scott J., Sanderson K. (2013). Building on the positives: A psychometric review and critical analysis of the construct of Psychological Capital. J. Occup. Organ. Psychol..

[48] Maggio D.I., Ginevra M., Nota L. (2021). The Role of Psychological Capital in Human Service Professionals’ Work Experiences. Eur. J. Investig. Health Psychol. Educ..

[49] Gom D., Lew T., Jiony M., Tanakinjal G., Sondoh S.J. (2021). The Role of Transformational Leadership and Psychological Capital in the Hotel Industry: A Sustainable Approach to Reducing Turnover Intention. Sustainability.

[50] Lei H., Leaungkhamma L., Le P.B. (2020). How transformational leadership facilitates innovation capability: The mediating role of employees’ psychological capital. Leadersh. Organ. Dev. J..

[51] Le B.P., Lei H., Phouvong S., Than T.S., Nguyen T.M., Gong J. (2018). Self-Efficacy and Optimism Mediate the Relationship between Transformational Leadership and Knowledge Sharing. Soc. Behav. Personal. Int. J..

[52] McColl-Kennedy J.R., Anderson R.D. (2002). Impact of leadership style and emotions on subordinate performance. Leadersh. Q..

[53] Salam A.H., Dumit N., Clinton M., Mahfoud Z. (2023). Transformational leadership and predictors of resilience among registered nurses: A cross-sectional survey in an underserved area. BMC Nurs..

[54] Azka G., Tahir M.Q., Syed T.H. (2011). Transformational leadership, employee engagement and performance: Mediating effect of psychological ownership. Afr. J. Bus. Manag..

[55] Bollen K., Pearl J., Morgan E.S. (2013). Eight myths about causality and structural equation models. Handbook of Causal Analysis for Social Research.

[56] Nazir O., Islam J.U. (2017). Enhancing organizational commitment and employee performance through employee engagement. South Asian J. Bus. Stud..

[57] Yuan Y., Kong H., Baum T., Liu Y., Liu C., Bu N., Wang K., Yin Z. (2022). Transformational leadership and trust in leadership impacts on employee commitment. Tour. Rev..

[58] Afsar B., Shahjehan A., Shah S.I., Wajid A. (2019). The mediating role of transformational leadership in the relationship between cultural intelligence and employee voice behavior: A case of hotel employees. Int. J. Intercult. Relat..

[59] Gooty J., Gavin M., Johnson P.D., Frazier M.L., Snow D.B. (2009). In the Eyes of the Beholder: Transformational Leadership, Positive Psychological Capital, and Performance. J. Leadersh. Organ. Stud..

[60] Yim H.-Y., Seo H.-J., Cho Y., Kim J. (2017). Mediating Role of Psychological Capital in Relationship between Occupational Stress and Turnover Intention among Nurses at Veterans Administration Hospitals in Korea. Asian Nurs. Res..

[61] Hair J.F., Babin B.J., Anderson R.E. (2019). Multivariate Data Analysis.

[62] Pearce C., Sims H. (2002). Vertical versus shared leadership as predictors of the effectiveness of change management teams: An examination of aversive, directive, transactional, transformational, and empowering leader behaviors. Group Dyn. Theory Res. Pract..

[63] Esteves T., Lopes M.P., Geremias R.L., Palma P. (2018). Calling for leadership: Leadership relation with worker’s sense of calling. Leadersh. Organ. Dev. J..

[64] Geremias R., Lopes M.P., Soares A. (2022). Psychological Capital Profiles and Their Relationship with Internal Learning in Teams of Undergraduate Students. Front. Psychol..

[65] Geremias R., Lopes M., Soares A. (2021). The influence of psychological capital on internal learning: The mediating role of the perceived team structure. RAE-Rev. De. Adm. De. Empresa.

[66] Fornell C., Larcker D.F. (1981). Evaluating Structural Equation Models with Unobservable Variables and Measurement Error. J. Mark. Res..

[67] Westland C.J. (2010). Lower bounds on sample size in structural equation modeling. Electron. Commer. Res. Appl..

[68] Richardson H.A., Simmering M.J., Sturman M.C. (2009). A Tale of Three Perspectives. Organ. Res. Methods.

[69] Podsakoff P., MacKenzie S.B., Lee J.-Y., Podsakoff N. (2003). Common method biases in behavioral research: A critical review of the literature and recommended remedies. J. Appl. Psychol..

[70] Williams L.J., McGonagle A.K. (2015). Four Research Designs and a Comprehensive Analysis Strategy for Investigating Common Method Variance with Self-Report Measures Using Latent Variables. J. Bus. Psychol..

[71] Baron R.M., Kenny D.A. (1986). The moderator–mediator variable distinction in social psychological research: Conceptual, strategic, and statistical considerations. J. Personal. Soc. Psychol..

[72] Sobel M.E., Leinhart E.S. (1982). Asymptotic intervals for indirect effects in structural equations models. Sociological Methodology.

[73] Ribeiro N., Gupta M., Gomes D., Alexandre N. (2021). Impact of psychological capital (PsyCap) on affective commitment: Mediating role of affective well-being. Int. J. Organ. Anal..

[74] Leach L. (2005). Nurse executive transformational leadership and organizational commitment. J. Nurs. Adm..

[75] Tang Y., Shao Y.-F., Chen Y.-J. (2019). Assessing the Mediation Mechanism of Job Satisfaction and Organizational Commitment on Innovative Behavior: The Perspective of Psychological Capital. Front. Psychol..

[76] Afshari L., Gibson P. (2016). How to increase organizational commitment through transactional leadership. Leadersh. Organ. Dev. J..

[77] Avolio B.J., Gardner W.L. (2005). Authentic leadership development: Getting to the root of positive forms of leadership. Leadersh. Q..

[78] Bahadir N. (2022). The Effect of Dark Leadership on Organizational Commitment: A Research in The Banking Sector. J. Bus. Res..

[79] Weymer A.S., Schuber J.K., Eskenazi A.S., Martins P.A. (2018). A contribuição de mulheres líderes no nível de comprometimento organizacional. Rev. De. Gestão Organ..

[80] Lin X., Zhu Y., Wang C., Wang F. (2021). Relationship among affective commitment, occupational stressors, and calling of psychiatrists in Shanghai. Medicine.

[81] Luthans F., Avey J., Clapp-Smith R., Li W. (2008). More evidence on the value of chinese workers’ psychological capital: A potentially unlimited competitive resource?. Int. J. Hum. Resour. Manag..

[82] Donaldson S.I., Villalobos J., Chen C.L. (2020). The Generalizability of HERO across 15 Nations: Positive Psychological Capital (PsyCap) beyond the US and Other WEIRD Countries. Int. J. Environ. Res. Public Health.

[83] Russo S.D., Stoykova P. (2015). Psychological Capital Intervention (PCI): A Replication and Extension. Hum. Resour. Dev. Q..

[84] Bligh M., Kohles J., Pearce C., Justin J., Stovall J. (2007). When the romance is over: Follower perspectives of aversive leadership. Appl. Psychol..

[85] Siraneh Y., Ololo S., Tsega G., Yitbarek K., Adamu A., Erchafo B., Hailu M., Woldie M. (2018). Level and Factors Associated with Professional Commitment of Health Professionals Providing Institutional Delivery Services in Public Health Facilities, Southwest Ethiopia. Ethiop. J. Health Sci..

